# CD4^+^ T Helper Cells Play a Key Role in Maintaining Diabetogenic CD8^+^ T Cell Function in the Pancreas

**DOI:** 10.3389/fimmu.2017.02001

**Published:** 2018-01-18

**Authors:** Gabriel Espinosa-Carrasco, Cécile Le Saout, Pierre Fontanaud, Thomas Stratmann, Patrice Mollard, Marie Schaeffer, Javier Hernandez

**Affiliations:** ^1^INSERM U1183, Institute for Regenerative Medicine and Biotherapy, University of Montpellier, Montpellier, France; ^2^Institute of Functional Genomics, CNRS, INSERM, University of Montpellier, Montpellier, France; ^3^Faculty of Biology, Department of Cell Biology, Physiology and Immunology, University of Barcelona, Barcelona, Spain

**Keywords:** autoimmunity, CD4 help, effector CD8^+^ T cells, type 1 diabetes, imaging, *in vivo*

## Abstract

Autoreactive CD8^+^ and CD4^+^ T cells have been assigned independent key roles in the destruction of insulin-producing beta cells resulting in type 1 diabetes. Although CD4 help for the generation of efficient CD8^+^ T cell responses in lymphoid tissue has been extensively described, whether these two cell populations cooperate in islet destruction *in situ* remains unclear. By using intravital 2-photon microscopy in a mouse model of diabetes, we visualized both effector T cell populations in the pancreas during disease onset. CD4^+^ T helper cells displayed a much higher arrest in the exocrine tissue than islet-specific CD8^+^ T cells. This increased arrest was major histocompatibility complex (MHC) class II-dependent and locally correlated with antigen-presenting cell recruitment. CD8^+^ T cells deprived of continued CD4 help specifically in the pancreas, through blocking MHC class II recognition, failed to maintain optimal effector functions, which contributed to hamper diabetes progression. Thus, we provide novel insight in the cellular mechanisms regulating effector T cell functionality in peripheral tissues with important implications for immunotherapies.

## Introduction

Type 1 diabetes (T1D) is an autoimmune disease characterized by progressive infiltration of pancreatic islets of Langerhans by immune cells and subsequent destruction of insulin-producing beta cells. Disease development reflects a failure of immune tolerance mechanisms that control self-reactive T cells (1). Although the exact composition of immune cells in diabetic lesions varies with time, T cells usually dominate in T1D patients (2). Both CD4^+^ helper and CD8^+^ cytotoxic T cells (CTLs) are present, and there is a large body of evidence indicating that both are key effectors in T1D development (2, 3). Indeed, most identified susceptibility loci contain candidate genes that are involved in T cell activation and regulation, and specific major histocompatibility complex (MHC) class II and, to a lesser extent, MHC class I haplotypes confer predisposition to diabetes development (1, 3).

Disease onset involves two sequential steps in anatomically distinct tissues. First, autoreactive T cells, which have evaded tolerance checkpoints, are activated in the lymph nodes draining the pancreas (pLN) by antigen-presenting cells (APCs) that display beta cell antigens (4, 5). At this stage, CD4^+^ T cells are required for efficient activation of CD8^+^ T cells (6–8). Second, activated effector T cells infiltrate the pancreas (9). While infiltrating effector CD4^+^ T cells are thought to contribute to beta cell death through macrophage activation (10), CD8^+^ T cells (11) are able to directly kill beta cells in an antigen-dependent manner (9, 12). In addition, both effector T cells engage in antigen-dependent contacts with APCs, which are required to maintain effector function over time in the pancreas, at least for CD8^+^ T cells (13, 14). These interactions appear to be crucial for the maintenance of cytotoxic properties in the periphery (15, 16).

Dynamics of T cell activation in secondary lymphoid organs, including pLN (17), have been extensively studied for over a decade in mouse models using two-photon microscopy. These studies have provided a framework for understanding the role of different patterns of dynamic interactions with APCs, leading to either T cell priming or tolerance induction (17, 18). Moreover, they highlighted the importance of cooperation between T cells in the generation of immune responses. In particular, recent studies have revealed a complex choreography of sequential interactions among APCs, CD4^+^, and CD8^+^ T cells in lymphoid organs during priming and the existence of a specialized dendritic cell (DC) subtype that may serve as a platform for CD4^+^ and CD8^+^ T cell cooperation (19, 20). Diabetogenic CD8^+^ or CD4^+^ T cell dynamics have been studied in pancreatic islets (9, 14, 21). However, visualizing T cell responses in deep organs such as the pancreas *in vivo* remains challenging and functional cooperation between CD8^+^ or CD4^+^ T cell populations in the pancreas during T1D remains unexplored.

In this study, we therefore set out to understand how effector CD8^+^ and CD4^+^ T cells cooperate in islet destruction during the onset of T1D. To allow this, intravital imaging approaches were applied directly to the pancreas in a mouse model of autoimmune diabetes, in which both CD8^+^ and CD4^+^ T cells are required to induce disease (6).

## Materials and Methods

### Mice

Mice were bred in SPF facility and housed in conventional facility during experimentation. To induce diabetes, we used the InsHA transgenic mouse model. Balb/c InsHA mice express the influenza virus hemagglutinin (HA) under the control of rat insulin promoter, driving its expression in pancreatic beta cells (22). Balb/c clone 4 TCR and HNT TCR transgenic mice express HA-specific MHC class I and class II restricted TCRs, respectively (23, 24). Naive clone 4 CD8^+^ and HNT CD4^+^ T cells adoptively co-transferred into sublethally irradiated InsHA mice undergo lymphopenia-induced proliferation and differentiate into memory-like cells (6). Under these conditions, HNT CD4^+^ T cells promote the further differentiation of clone 4 CD8^+^ T cells into effectors in the draining lymph nodes of the pancreas, their migration to the pancreas, and onset of autoimmune diabetes (6). InsHA (22), clone 4 TCR (23), and HNT TCR (24) were kindly provided by L. A. Sherman (The Scripps Research Institute, San Diego, CA, USA). For imaging purposes, fluorescent labels were introduced in beta cells by crossing InsHA mice with RIP-mCherry mice (25), and clone 4 TCR and HNT TCR transgenic mice were crossed with actin-GFP and actin-CFP transgenic mice, respectively. RIP-mCherry mice (25) were provided by P. Le Tissier and I. C. Robinson (National Institute of Medical Research, London, UK), and β-actin-GFP and β-actin-CFP mice were from the Jackson Laboratory. InsHA, clone 4 TCR, and HNT TCR were backcrossed with BALB/c Thy1.1^+/+^ mice for >15 generations, while RIP-mCherry, β-actin-GFP, and β-actin-CFP mice were backcrossed with C57BL/6 mice for >15 generations. F1 clone 4 TCR Thy1.1 × actin-GFP (clone 4-GFP), F1 HNT TCR Thy1.1 × actin-CFP (HNT-CFP), and F1 InsHA × RIP-mCherry mice on BALB/c × C57BL/6 background 10–16 weeks of age were used. More than 98% of the CD8^+^ T cells from clone 4-GFP mice were Vβ8.2^+^, and 93% of the CD4^+^ T cells from HNT-CFP mice were Vβ8.3^+^.

### T Cell Isolation, Adoptive Transfer, and Diabetes Monitoring

Naive CD8^+^ T cells from clone 4 TCR Thy1.1 × β-actin-GFP and CD4^+^ T cells from HNT TCR Thy1.1 × β-actin-CFP F1 mice were prepared from LN and spleen using T cells isolation kits (Dynabeads, Thermo Fisher Scientific). Equal numbers (2–3 × 10^6^ cells/recipient) of CD8^+^ and CD4^+^ T cells were injected i.v. into InsHA × RIP-mCherry mice sublethally irradiated (4.5 Gy) 24 h before in a therapeutic irradiator (Varian). Some mice received either CD8^+^ or CD4^+^ T cells (2–3 × 10^6^ cells/recipient) separately. Recipient mice blood glucose levels were monitored using a glucometer (AccuCheck). All experiments used normoglycemic mice, except for diabetes-onset kinetics and survival analyses, in which diabetic mice (>300 mg/dl of blood glucose for 2 consecutive days) were monitored daily and euthanized at first signs of distress.

### Surgery and Intravital Imaging

Mice pancreas was exteriorized by surgery as described (25). Briefly, animals were anesthetized by injection of ketamine/xylazine (0.1/0.02 mg/g). Respiration was controlled by tracheotomy to limit tissue movement. The pancreas was gently maneuvered onto a metallic stage covered with a soft polymer (Bluesil) and pinned using stainless steel minutien insect pins (tip = 0.0125 mm). The tissue was continuously superfused with a NaCl 0.9% heated to 37°C. Fluorescent lymphocytes and beta cells were visualized using a multiphoton microscope (Zeiss 7MP) adapted with a long-working distance objective M Plan Apo NIR × 20, 0.4 NA (Mitutoyo). Excitation was achieved using a Ti:Sapphire Chameleon Laser (Coherent) tuned to either 820 nm (mCherry and mCherry-GFP-CFP excitation), 850 nm (rhodamine-GFP-CFP), 880 nm (GFP-CFP), or 910 nm (rhodamine-GFP). Emitted fluorescence was captured using GaAsP photomultiplier tubes at 460–500 nm for CFP, 500–550 nm for GFP, and 610–700 nm for mCherry and rhodamine. Surface islets (<100 µm in depth) were identified using mCherry or by light contrast. Tissue viability was verified by rhodamine-dextran i.v. injection and detection of blood flow as described (25).

### Image Data Analysis

Stacks 150–250 µm thick (Z steps of 3 µm) were acquired every 30 s to 1 min for 10–20 min. Movies were stabilized using Huygens Essential (SVI). Only areas with similar infiltration levels were compared. Fields presenting <20 and >500 T cells/0.05 mm^3^ (imaging stack volume) were excluded from analysis. Mild and severe infiltration areas were defined as presenting less or more than 150 T cells/0.05 mm^3^, respectively. Measurements were performed in at least three independent experiments. Average velocities and instantaneous speeds were obtained using Imaris (Bitplane). Arrest coefficients (percentage of time points at instantaneous velocities below 3 µm/min) were calculated using a MATLAB routine (26). T cell coordinates obtained using Imaris were imported in MATLAB to generate graphs of XY projections of T cell tracks. Tracks lasting <4 min were excluded. No exclusion was made based on velocity. Cellular contacts were manually quantified and verified by rotations in Z using Imaris.

### Flow Cytometry

For T cell phenotyping, pancreas, pLN, and peripheral LN were processed and stained as described (6). The mAbs used were as follows: anti-CD4-BV711, anti-CD4-FITC, anti-CD8a-BV786, anti-CD8a-FITC, anti-Thy1.1-PerCP, anti-CD62L-APC, anti-INFγ-PE, anti-CD45RB-PE, and anti-CD107a-PE (BD PharMingen); anti-CD25-APC-eFluor780, anti-Granzyme B-PE, anti-KLRG1-PE-Cy7, and anti-FoxP3-PerCP-Cy5.5 (eBioscience). Cells were analyzed on a FACSCanto II or a LSR Fortessa apparatus using Diva software (BDB). Isotype-matched antibodies were used as controls. Intracellular Granzyme B and Foxp3 staining was performed using the Fixation and Permeabilization Kit (eBioscience). Intracellular IFNγ and IL-2 staining was performed after 5 h restimulation with HA peptide using the Cytofix/Cytoperm Kit (BD PharMingen) (6).

### *In Vivo* Antibody Treatment

Anti-MHC class II (I-A/I-E) (clone M5/114, BioXcell) and isotype control antibody [rat IgG2b/anti-KLH (BioXcell)] were either injected through a jugular catheter on day 8 after T cell transfer >1 h before imaging (200 μg/animal) or i.p. on days 8 (200 µg) and 9 (100 µg) after T cell transfer and mice were euthanized at day 10 for FACS analysis. For survival analysis, mice received 200 µg i.p. on day 8 and 100 µg every 3 days until sacrifice.

### Confocal Imaging

Pancreata were fixed in 4% paraformaldehyde and sliced on a vibratome (Leica) (100 µm), or snap-frozen and cryostat-sectioned slices (20 µm) were fixed with 1.5% paraformaldehyde. Immediate fixation was necessary to preserve CFP and GFP fluorescence. Antibody labeling was done as described previously (27). The antibodies used were as follows: hamster anti-CD11c (N418, eBioscience), rat anti-F4/80 (MCA4976, BioRad), rabbit anti-insulin (Cell Signaling), and rat anti-endomucin (Santa Cruz Biotechnology). Nuclei were labeled using dapi (Sigma). Images were acquired using a Zeiss LSM 780 confocal microscope and analyzed using Imaris (Bitplane), Volocity (Perkin Elmer), and ImageJ (NIH). One to four slices were randomly selected from at least three animals per group. For quantification of infiltrated islets, four slices were randomly selected from pancreas from at least three animals per group, and all islets present were analyzed (>60 islets/mouse). For quantification of T cells–APCs contacts, four slices were randomly selected from at least three animals per group, and all cells present in a minimum of 10 different confocal images (700 μm × 700 μm × 20 μm) were analyzed.

### Statistical Analysis

Values are represented as means ± SEM. Statistical tests were performed using GraphPad Prism. Normality was tested using D’Agostino-Pearson test, and comparisons were made using either unpaired Student’s *t*-test or two-tailed Mann–Whitney *U*-test, as appropriate. Multiple comparisons were made using one-way ANOVA followed by Bonferroni’s *post hoc* test. *P* values were considered significant at *P < 0.05, **P < 0.01, ***P < 0.001, and ****P < 0.0001.

## Results

### Simultaneous Intravital Imaging of Islet Antigen-Specific CD8^+^ and CD4^+^ T Cell Behavior

**Figure 1:**
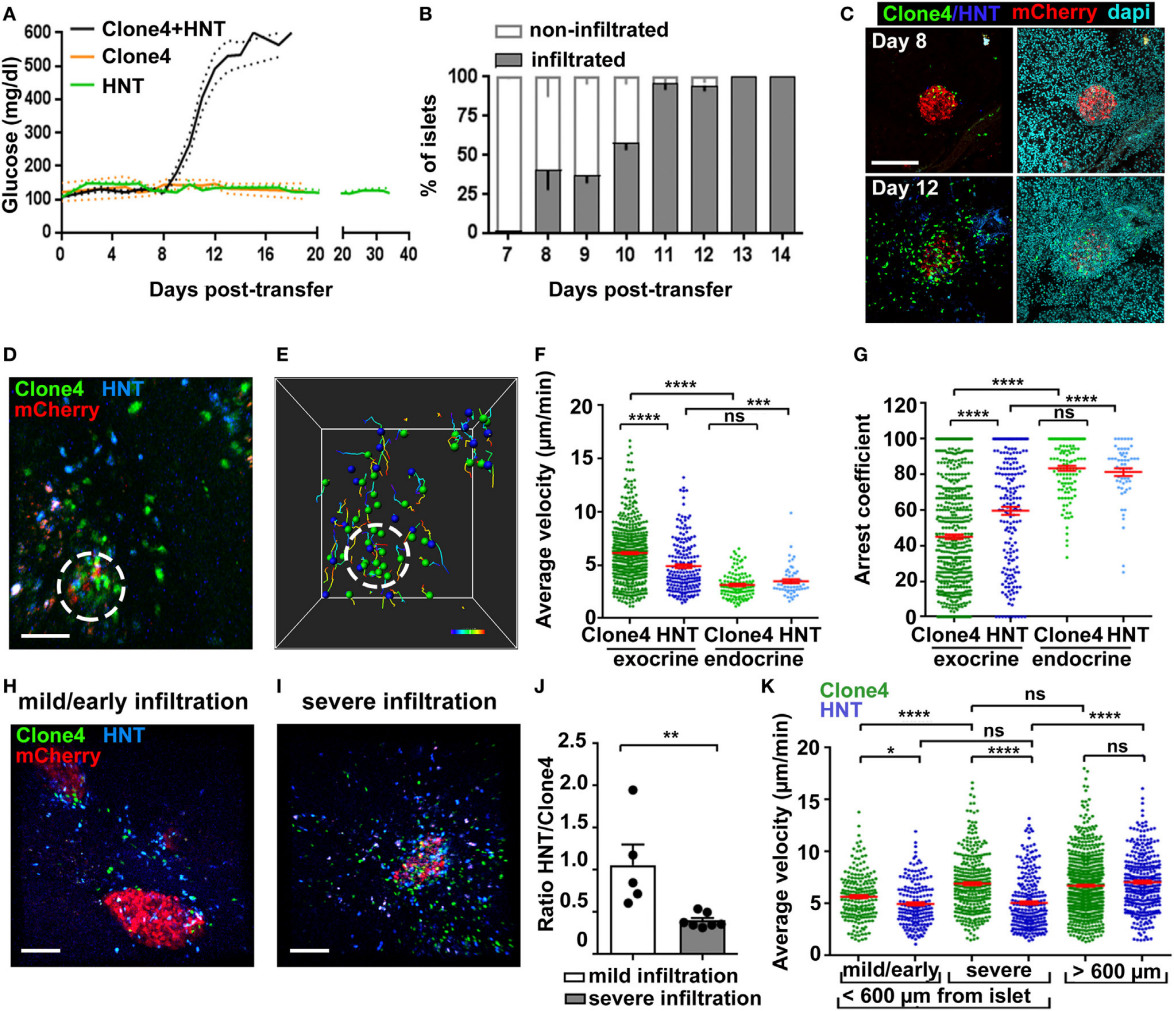
Infiltration and *in vivo* motility of islet antigen-specific CD8^+^ and CD4^+^ T cells in the pancreas. **(A)** Blood glucose levels of irradiated InsHA-mCherry mice as function of days posttransfer of either HNT-GFP CD4^+^, clone 4-GFP CD8^+^, or clone 4-GFP CD8^+^ and HNT-CFP CD4^+^ T cells (*n* = 3–48 mice/condition). **(B)** Percentage of infiltrated islets in irradiated InsHA-mCherry mice transferred with clone 4-GFP CD8^+^ and HNT-CFP CD4^+^ T cells as function of days posttransfer (*n* = 3–7 mice/day, >150 islets/mouse). **(C)** Representative confocal images of pancreas at day 8 and 12 posttransfer of mice described in **(B)** (scale: 200 µm, 25 µm Z-projection). **(D)** Still image from a representative movie in the pancreas of a mouse described in **(B)** at day 8 posttransfer (scale: 50 µm, Z-projection of 200 µm) (Video S1 in Supplementary Material). Green, CD8^+^; blue, CD4^+^; red, beta cells. Islet is circled. **(E)** T cells tracks from field in **(D)**, color-coded as function of time. Total duration: 18.3 min. **(F,G)** Average T cell velocities **(F)** and arrest coefficients **(G)** (% time points spent at <3 µm/min for each track) in exocrine (outside of circle around mCherry signal) and endocrine (inside of circle) tissues at day 8 posttransfer (*n* = 3–5 mice/condition; 1–2 movies/mouse with an islet in the field, one-way ANOVA). Dots correspond to individual T cells. **(H,I)** Still images from representative movies of areas of a single pancreas at day 8 posttransfer presenting either mild **(H)** (<150 CFP and GFP cells/0.05 mm^3^) or severe **(I)** (>150) infiltration (scale: 100 µm, 160 µm Z-projection) (Video S3 in Supplementary Material). **(J)** Ratio of CFP to GFP cells in exocrine tissue (*n* = 3–5 mice/condition; 1–2 movies/mouse, one-way ANOVA). Dots correspond to individual movies. **(K)** Average T cell velocities at day 8 posttransfer in either mildly or severely infiltrated areas close to islets (<600 µm) or away (>600 µm) (*n* = 3–5 mice/condition; 1–2 movies/mouse, one-way ANOVA) (see also Video S5 in Supplementary Material). Values are represented as mean ± SEM.

To study the interplay of effector CD8^+^ and CD4^+^ T cells during pancreas infiltration leading to beta cell destruction, we utilized InsHA transgenic mice that express the influenza HA antigen in beta cells. On adoptive transfer into InsHA mice, both HA-specific TCR transgenic clone 4 CD8^+^ and HNT CD4^+^ T cells (MHC class I or II restricted, respectively) are required to induce diabetes under inflammatory conditions (8). In this model, CD4^+^ T helper cells are essential for CD8^+^ T cell differentiation into CTLs in the pLN (6). Here, fluorescent labels were introduced into beta cells (mCherry), clone 4 CD8^+^ (GFP), and HNT CD4^+^ (CFP) T cells. As expected (6), naive clone 4-GFP CD8^+^ T cells co-transferred with HNT-CFP CD4^+^ T cells into sublethally irradiated InsHA-mCherry hosts expanded after day 6 posttransfer, differentiated into CTL in the pLN and migrated to the pancreas, together with HNT-CFP CD4^+^ T cells (Figures S1A–E in Supplementary Material). Of note, from 77 to 93% of GFP and CFP cells found in InsHA-mCherry hosts corresponded to Thy1.1^+^ CD8^+^ and Thy1.1^+^ CD4^+^ T cells, respectively (Figure S1F in Supplementary Material). This resulted in autoimmune diabetes in all mice tested (Figure 1A). In the absence of HNT-CFP CD4^+^ T cells, clone 4-GFP CD8^+^ T cells did not differentiate into effectors in the pLN, as evidenced by the lack of expression of CD25, Granzyme B, and KLRG1 as well as the high expression of CD62L, and were unable to migrate to the pancreas (Figure 1A; Figures S2A–C in Supplementary Material). However, clone 4-GFP CD8^+^ T cells from all LN were able to secrete low amounts of IFNγ (Figure S2B in Supplementary Material), a general phenomenon observed when naive T cells are transferred into irradiated mice (28). In contrast, both phenotype and capacity of HNT-CFP CD4^+^ T cells to migrate to the pancreas were independent of clone 4-GFP CD8^+^ T cells (Figures S1A,D,E and S2D–F in Supplementary Material). However, HNT-CFP CD4^+^ T cells alone did not induce diabetes (Figure 1A), predominantly remaining in a peri-insulitic disposition before clearance (Figure S3 in Supplementary Material). The HNT-CFP CD4^+^ T cell population contained a small subset of FoxP3^+^ CD25^+^ cells whose proportion, 4–5%, also remained constant regardless of the presence or absence of donor CD8^+^ T cells or the anatomical location (Figures S1C and S2C in Supplementary Material). Taken together, these results demonstrate that our autoimmune diabetes model in a mixed background resembles closely to that previously described in a Balb/c background (6).

Analysis of islet infiltration in InsHA-mCherry mice co-transferred with clone 4-GFP and HNT-CFP T cells demonstrated progressive insulitis (Figures 1B,C), which coincided with rapid and synchronized increase in blood glucose levels from day 8 posttransfer (Figure 1A). Different lobes of the pancreas presented varying infiltration levels at day 8, and infiltrated islets were irregularly distributed. This reflects the spatiotemporal heterogeneity of the autoimmune attack, as previously reported in other mouse models (14, 29) and human patients (2, 30). We therefore analyzed T cell dynamics at that particular time point to cover a broad range of islet infiltration levels. By using a recently described two-photon microscopy approach (25), we were able to visualize beta cells, clone 4-GFP, and HNT-CFP T cells in prediabetic InsHA-mCherry mice (Figure 1D; Video S1 in Supplementary Material) and track T cell motility (Figure 1E). Both T cell types accumulated in islets and surrounding exocrine tissue (Figure 1D). Clone 4-GFP CD8^+^ T cells displayed significantly higher average velocity and lower arrest coefficient (percentage of time points at velocity <3 µm/min) than HNT-CFP CD4^+^ T cells (7 vs. 5 µm/min, and 47 vs. 59%, respectively) in exocrine tissue (Figures 1F,G). By contrast, both cells types behaved similarly within islets and displayed lower average velocity (~3.5 µm/min) and higher arrest coefficients (>80%) (Figures 1F,G). In correlation with their higher arrest, most clone 4-GFP CD8^+^ T cells within islets engaged in contacts with beta cells (Video S2 in Supplementary Material). Although these interactions were presumably leading to beta cell destruction, the described (9) rare fluorescent tag extinction in beta cells was not observed here over the imaging time course used.

Since variable numbers of T cells could be observed in imaging fields at day 8, we evaluated separately T cell behavior in mildly infiltrated (<150 cells/0.05 mm^3^) and severely infiltrated (>150 cells/0.05 mm^3^) areas of the exocrine tissue (outside islets) (Figures 1H,I; Video S3 in Supplementary Material). Mild infiltration was mainly observed in close proximity to islets (i.e., the exocrine tissue directly adjacent to an islet in the imaging field: <200 µm in Z, or <600 µm in X/Y). Therefore, heavily infiltrated areas with similar location parameters were chosen for comparison. We found that the shift from mild to severe infiltration in the exocrine tissue around islets was marked by a change in the CFP to GFP ratio from 1:1 to 1:2.5 (Figure 1J), possibly reflecting a differential influx of CD8^+^ T cells as infiltration progresses (2, 29) and/or their proliferation in the pancreas (16), as could be observed here *in vivo* (Video S4 in Supplementary Material, total of three events observed over 6.25 h total elapsed movie time). In addition, we found that clone 4-GFP T cell motility in the exocrine tissue increased as a function of infiltration (Figure 1K). By contrast, HNT-CFP T cell motility was equivalent in mildly and heavily infiltrated areas (Figure 1K). Finally, we assessed whether the distance to the source of cognate antigen affected T cell motility. For this purpose, we imaged infiltrated fields in which no islet was visible. In this case, both clone 4-GFP CD8^+^ and HNT-CFP CD4^+^ T cells migrated with similar average velocities (distance >600 µm from an islet) (Figure 1K; Video S5 in Supplementary Material). However, HNT-CFP CD4^+^ T cells displayed increased motility compared to those in close vicinity to islets. To test whether migratory behavior of HA-specific T cells was antigen driven, we co-transferred either non-antigen-specific CD4^+^ or CD8^+^ T cells, in equal numbers, along with clone 4 CD8^+^ and HNT CD4^+^ T cells. However, non-HA-specific T cells, despite of being activated, failed to be recruited to the pancreas by day 8 posttransfer in numbers sufficient for analysis (data not shown). Thus, the pancreas environment seems less permissive than other inflamed peripheral tissues like the brain to bystander effector T cell recruitment (26). Taken together, these results show that HA-specific CD8^+^ and CD4^+^ T cells display different kinetics of infiltration and migration in the exocrine tissue, suggesting that divergent mechanisms may be involved in the regulation of their motilities/arrest.

### Islet Antigen-Specific CD4^+^ T Cell Arrest Correlates with Local Recruitment of Myeloid Cells

**Figure 2:**
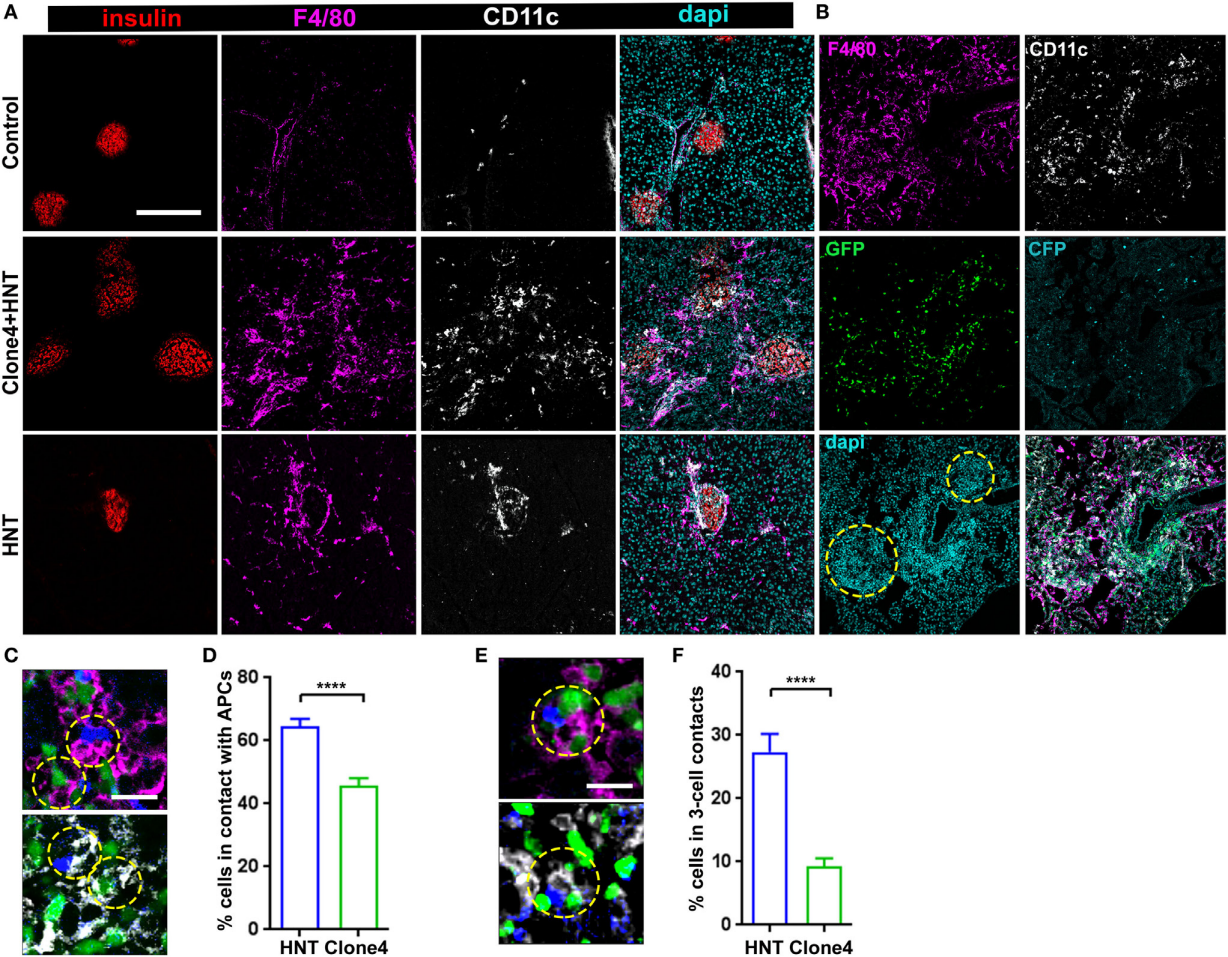
Islet antigen-specific CD4^+^ T cell arrest correlates with localized recruitment of myeloid cells at infiltration sites. **(A)** Representative confocal images of pancreas from irradiated non-transferred, irradiated co-transferred with clone 4-GFP CD8^+^ and HNT-CFP CD4^+^, and irradiated transferred with HNT-CFP CD4^+^ InsHA-mCherry mice at day 8 posttransfer stained with the indicated mAbs (scale: 200 µm, Z-projection of 10 µm). **(B)** Representative confocal images of the pancreas from an irradiated InsHA-mCherry mouse at day 8 posttransfer of HNT-CFP CD4^+^ and clone 4-GFP CD8^+^ T cells (scale: 200 µm, Z-projection of 6 µm). Islets identified by dense DAPI staining are circled. **(C)** Representative confocal images of pancreas from mice described in **(B)** at day 8 posttransfer (scale 20 µm, single Z-plane) depicting HNT-CFP CD4^+^ (blue) and clone 4-GFP CD8^+^ (green) cells in contact with an F4/80^+^ (purple, top) or CD11c^+^ (white, bottom) cell. Cells in contact are circled. **(D)** Percentage of HNT-CFP CD4^+^ and clone 4-GFP CD8^+^ T cells in contact with F4/80^+^ and CD11c^+^ cells in the exocrine tissue (*n* = 3 mice, *P* < 0.0001, Mann–Whitney *U*-test). **(E)** Representative confocal images of pancreas from mice described in **(B)** at day 8 posttransfer (scale 20 µm, single Z-plane) depicting a three-cell contact (HNT/APC/clone 4) with a F4/80^+^ (purple, top) or CD11c^+^ (white, bottom) cell. Cells in contact are circled. **(F)** Percentage of HNT-CFP CD4^+^ and clone 4-GFP CD8^+^ T cells forming three-cell contacts with CD11c^+^ APCs in exocrine tissue (*n* = 3 mice, *P* < 0.0001, Mann–Whitney *U*-test). Values are represented as mean ± SEM.

An intriguing feature of HA-specific T cell motility was that HNT-CFP T cells spent significantly more time arrested than clone 4-GFP T cells in the exocrine tissue in islet vicinity. Since CD4^+^ T cell arrest is mostly driven by MHC class II-dependent interactions with APCs (18, 31), we analyzed leukocyte recruitment accompanying pancreas infiltration. As expected (32), while a small population of resident macrophages and DCs could be detected in control irradiated non-transferred mice (Figure 2A), clone 4-GFP and HNT-CFP T cell transfer promoted the recruitment of F4/80^+^ and CD11c^+^ cells (Figure 2A). Notably, HNT-CFP CD4^+^ T cells transferred alone were able to induce such recruitment (Figure 2A). Recruited MHC class II^hi^ APCs mainly corresponded to DCs and macrophages (Figure S4 in Supplementary Material). While the majority of recruited DCs (62%) were from the CD11c^+^ CD103^+^ lineage, CD11c^+^ CD11b^+^ DCs were also present, as well as F4/80^+^ CD11b^hi^ Ly6c^hi^ macrophages. APC recruitment coincided with T cell infiltration (Figure 2B). Overall, a large proportion of HNT-CFP and clone 4-GFP T cells were in close apposition to F4/80^+^ and CD11c^+^ APCs (>60 and >40%, respectively) (Figures 2C,D). This suggests that different proportions of CD4^+^ and CD8^+^ T cells may interact with APCs at a given time point and could explain differences in T cell arrest in the exocrine tissue. Finally, about 27% of HNT-CFP CD4^+^ and 9% of clone 4-GFP CD8^+^ T cells, respectively, were involved in three-cell type CD4^+^/CD8^+^/APCs contacts (Figures 2E,F). These findings suggest that three-cell type contacts may occur *in vivo*.

### Effector CD4^+^ T Cell Help Support Diabetogenic CTL Effector Function in the Pancreas

**Figure 3:**
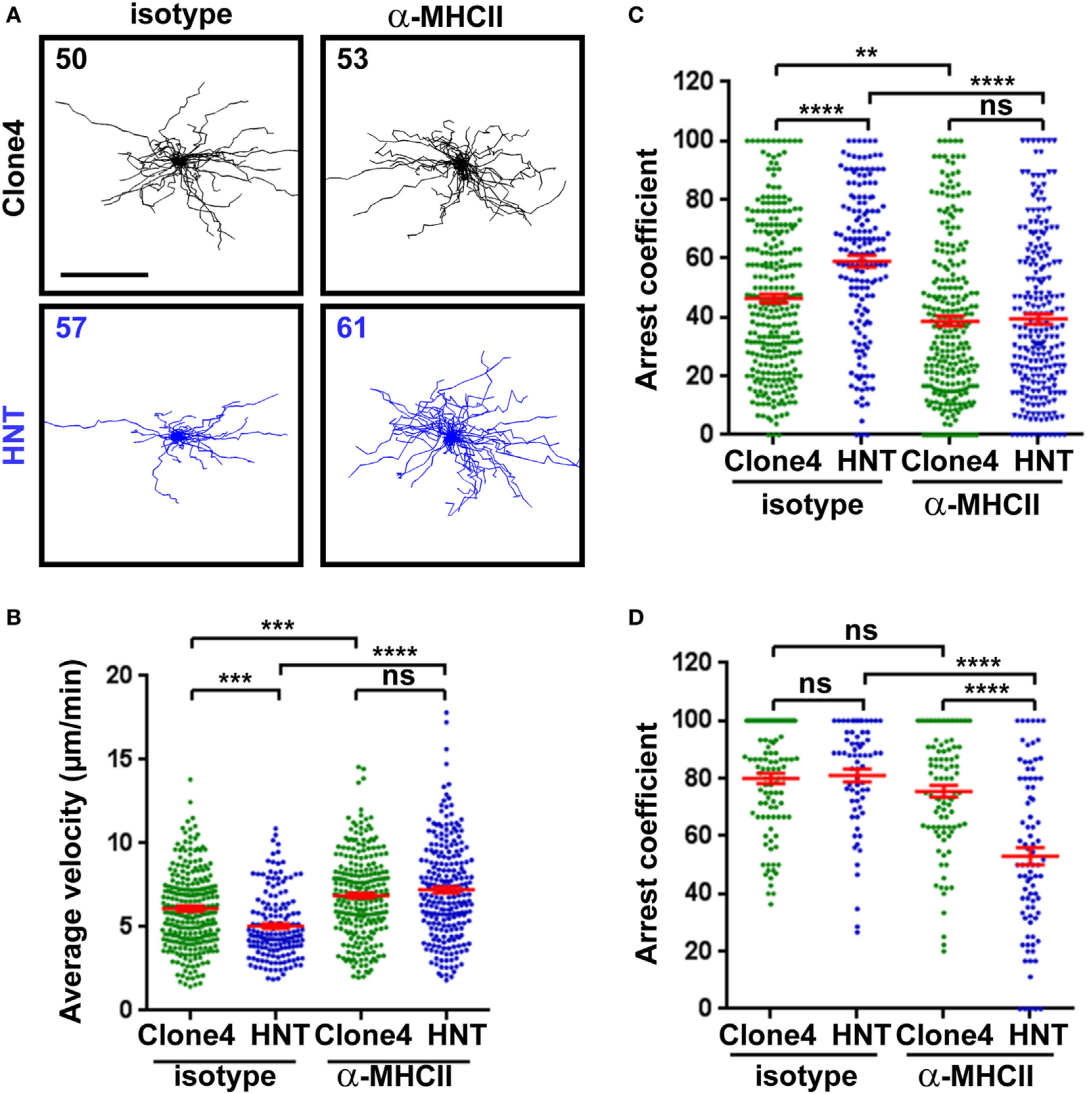
Major histocompatibility complex (MHC) class II-dependent CD4^+^ T cell arrest in the pancreas. **(A)** Irradiated InsHA-mCherry mice transferred with clone 4-GFP CD8^+^ and HNT-CFP CD4^+^ T cells were subjected to intravital microscopy on day 8. XY projections of T cell tracks over 17.5 min are depicted. Recording started >1 h following isotype-matched control or anti-MHCII antibody i.v. injection (scale: 100 µm) (Video S6 in Supplementary Material). Numbers indicate track numbers in movies. **(B)** Average T cell velocities in the exocrine tissue at day 8 posttransfer (*n* = 3 mice/condition; 1–3 movies/mouse, one-way ANOVA). **(C,D)** Arrest coefficients in exocrine **(C)** or endocrine **(D)** tissues at day 8 (*n* = 3 mice/condition; 1–3 movies/mouse in areas with comparable levels of infiltration, one-way ANOVA). Dots correspond to individual T cell tracks.

Recruited APCs may be able to present HA-derived peptides in the pancreas and mediate antigen-specific interactions with effector T cells (13, 14, 32). To investigate whether CD4^+^ T cell/APC interactions modulate effector CD8^+^ T cell function in the pancreas, we treated InsHA-mCherry mice with either anti-MHC class II or control mAb prior to imaging. Motility of HNT-CFP CD4^+^ T cells was greatly affected, and arrest coefficients in exocrine and endocrine tissues were significantly decreased (Figures 3A–D; Video S6 in Supplementary Material). These results indicate that MHC class II blockade efficiently prevented HNT-CFP CD4^+^ T cell lasting arrest on APCs.

**Figure 4:**
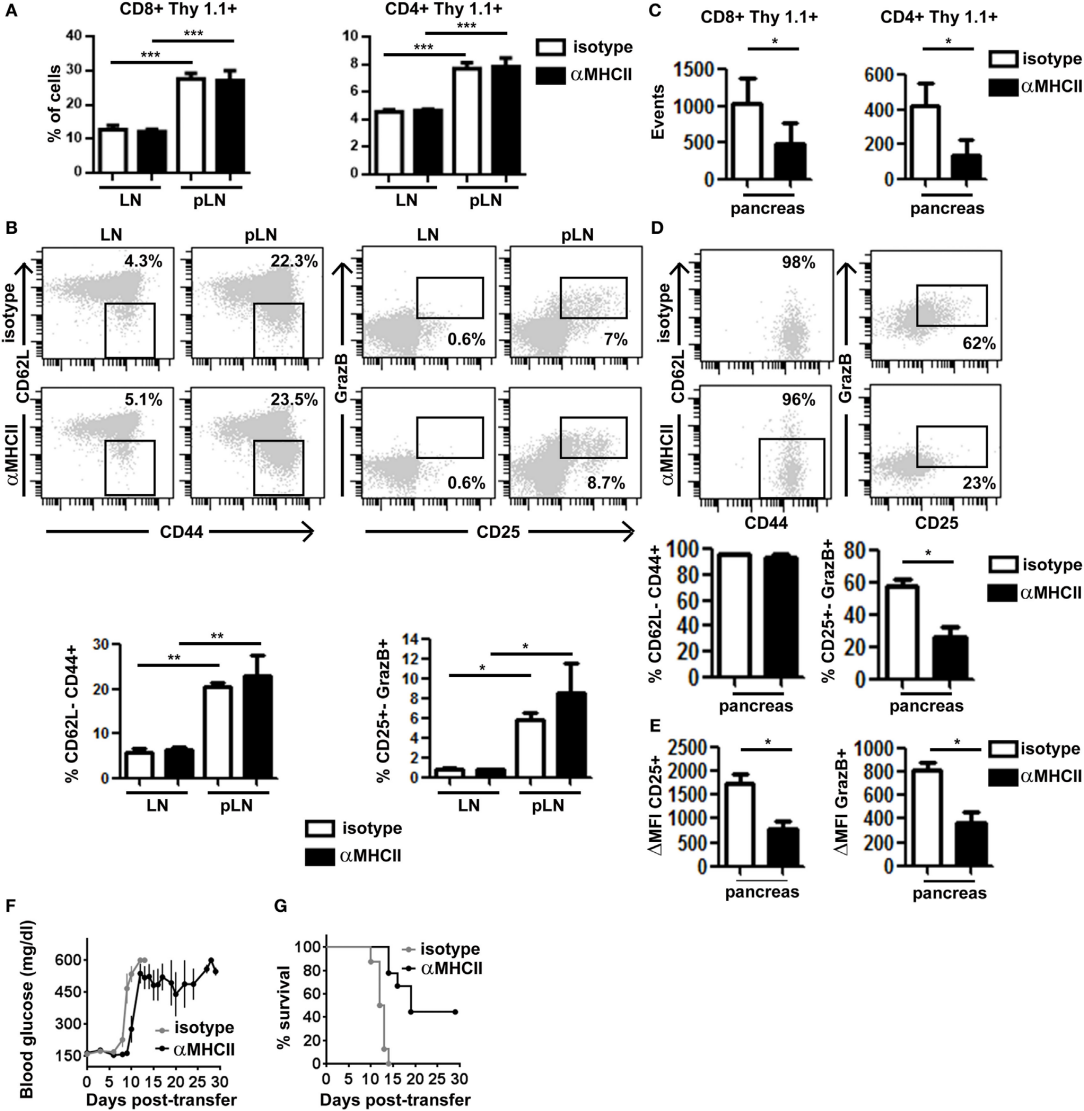
Major histocompatibility complex (MHC) class II-dependent CD4^+^ T cell help supports CD8^+^ T cell effector function in the pancreas. Irradiated InsHA-mCherry mice transferred with clone 4-GFP CD8^+^ and HNT-CFP CD4^+^ T cells were treated on days 8 and 9 with either anti-MHCII- or isotype-matched control mAb and sacrificed on day 10. **(A)** Detection of Thy1.1^+^ donor clone 4-GFP CD8^+^ and HNT-CFP CD4^+^ T cells in LN and pLN. Values represent mean ± SEM of the percentages of donor T cells in singlets living lymphocytes (*n* = 5 mice/group, Mann–Whitney *U*-test). **(B)** Phenotype of CD8^+^ Thy1.1^+^ donor lymphocytes in the LN and pLN of representative individual mice. Graphs depict percentages of different subpopulations of donor CD8^+^ T cells. Values correspond to mean ± SEM (*n* = 4 mice/group, Mann–Whitney *U*-test). **(C)** Detection of Thy1.1^+^ donor clone 4-GFP CD8^+^ and HNT-CFP CD4^+^ T cells in the pancreas. Values represent mean ± SEM of total detected events from individual pancreas (*n* = 5 mice/group, Mann–Whitney *U*-test). **(D)** Phenotype of clone 4-GFP CD8^+^ Thy1.1^+^ donor lymphocytes in the pancreas of representative individual mice. Graphs depict percentages of different subpopulations of donor clone 4-GFP CD8^+^ T cells in the pancreas. Values correspond to mean ± SEM (*n* = 3–5 mice/group, Mann–Whitney *U*-test). **(E)** Graphs depict the mean fluorescence intensity (MFI) of CD25 and granzyme B (GrazB) minus that of matched isotype control staining in the CD8^+^ Thy1.1^+^ CD25^+^ GrazB^+^ T cell subpopulations described in **(D)**. Values represent mean ± SEM (*n* = 3–5 mice per group, Mann–Whitney *U*-test). Data from one representative experiment of two, Mann–Whitney *U*-test. **(F)** Irradiated InsHA-mCherry mice transferred with clone 4-GFP CD8^+^ and HNT-CFP CD4^+^ T cells were treated from day 8 posttransfer every 3 days with either anti-MH II- or isotype-matched control antibody. Blood glucose levels over time post-T cell transfer, mean ± SEM (*n* = 9 mice/group, two independent experiments). **(G)** Survival curve of mice described in **(F)**.

Second, we evaluated the impact of preventing effector CD4^+^ T cell/APC contacts on CTL function in the pancreas. Mice co-transferred with clone 4-GFP and HNT-CFP T cells were treated with either anti-MHC class II or isotype control, mAbs at a time when effector T cells had already been generated in the pLN and started infiltrating the pancreas (days 8–9 after transfer). At day 10, we found equivalent numbers of HNT-CFP CD4^+^ and clone 4-GFP CD8^+^ T cells in peripheral LN and pLN of both control and treated mice (Figure 4A), indicating that blocking antibody treatment at that time point did not prevent antigen-driven activation of HA-specific T cells in the pLN. In fact, comparable effector clone 4-GFP CD8^+^ T cells were generated in the pLN of treated and control mice (Figure 4B). Not surprisingly, we found significantly less HNT-CFP CD4^+^ and clone 4-GFP CD8^+^ T cell infiltration in the pancreas of treated mice (Figure 4C). CD4^+^ T cell extravasation has been shown to be antigen dependent in the pancreas (32), and CD8^+^ T cell recruitment is favored by CD4^+^ T cells (16). Thus, influx of effector T cells from day 8 may be impaired, and cells found at day 10 may correspond to those that had already infiltrated the pancreas at day 8. Strikingly, important differences were observed in expression of markers linked to effector functions in clone 4-GFP CD8^+^ T cells (Granzyme B and CD25) (Figure 4D), as well as in their potential to secrete IFNγ (Figure S5A in Supplementary Material). Although in control mice, 62% of infiltrating CTLs were CD25^+^ GranzymeB^+^ and 56% produced IFNγ after *in vitro* restimulation, the percentage of bona fide effectors was reduced to <23% in anti-MHC class II-treated mice (Figure 4D; Figure S5A in Supplementary Material). Furthermore, the remaining CD8^+^ CD25^+^ GranzymeB^+^ T cells expressed those markers at lower levels than controls (Figure 4E). Notably, HNT-CFP CD4^+^ T cells also displayed reduced effector potential in the absence of antigenic stimulation, as evidenced by a decrease in their ability to produce IL-2 and IFNγ (Figure S5B in Supplementary Material). Thus, this indicates that help mediated by beta cell antigen-specific CD4^+^ T cells is required to maintain the functionality of diabetogenic CTL in the pancreas.

Finally, we evaluated whether this decline in CTL effector potential could impact disease progression by treating transferred InsHA-mCherry mice with anti-MHC class II from day 8 post-T cell transfer, every 3 days, for a 30-day period. All control mice became rapidly diabetic and had to be sacrificed (Figure 4F). Conversely, anti-MHC class II treatment delayed onset of hyperglycemia (Figure 4F). Strikingly, anti-MHC class II-treated mice, even with elevated blood glucose levels, remained healthy for longer periods and 44% survived until the end of the experiment (Figure 4G). Although the initial delay in the onset of hyperglycemia may result from the fact that fewer T cells infiltrate the pancreas, ulterior stabilization of blood glucose levels likely reflects the loss of effector function by diabetogenic CTL.

## Discussion

Dynamic influxes of different immune cell types in pancreatic islets lead to beta cell destruction (2, 29). While CD8^+^ and CD4^+^ T lymphocytes usually represent the dominant immune cell populations and independently play a key role in beta cell killing (9, 33), whether they are able to collaborate in islet destruction remained unknown. By using two-photon microscopy *in vivo* to visualize HA-specific CD8^+^ and CD4^+^ T cells in the pancreas of mice expressing HA in beta cells, we found that CD4^+^ T cells displayed MHC class II-dependent increased arrest in the exocrine tissue. CD4^+^ T cell arrest locally correlated with APC recruitment, which infiltrating CD4^+^ T cells were sufficient to trigger. Finally, infiltrated effector CD8^+^ T cells deprived of continued help through anti-MHC class II antibody treatment failed to maintain optimal effector functions specifically in the pancreas, which, together with reduced effector T cell infiltration, hampered diabetes progression. These results support a crucial role for effector CD4^+^ T helper cells in sustaining effector functions of CTLs at target sites.

In accordance with previous studies (14, 29), insulitis was heterogeneous. Notably, islets infiltrated by a few T cells contained both CD8^+^ and CD4^+^ T cells, suggesting that timing of infiltration is similar for both T cell types, albeit in different ratio. Both T cell populations migrated outside islets at average velocities similar to those described for T cells in peripheral tissues (26, 34). Their motility increased with infiltration levels (14), and arrest in islets was high, consistent with antigen recognition (9). By contrast with CD8^+^ T cells, CD4^+^ T cells presented a marked arrest in the exocrine tissue, correlating with recruitment of both DCs and macrophages. Local recruitment of APCs may serve different purposes. First, recruited macrophages may directly participate in beta cell killing (35). Second, recruited APCs may phagocytose killed cells, promoting beta cell antigen presentation and amplifying T cell activation. Finally, local aggregation of APCs may restrict T cell migration to favor induction or maintenance of effector functions and CTL proliferation (15, 36, 37).

Long-lasting CD8^+^ T cell responses are dependent on CD4 help in non-lymphoid tissues (16, 38). Evidence points to DCs as key mediators in this process. First, most MHC class II-expressing cells in the exocrine tissue here correspond to CD103^+^ DCs. Second, both beta cell antigen-specific CD8^+^ and CD4^+^ T cells have been independently shown to engage in antigen-mediated interactions with DCs (13, 14). Finally, anti-MHCII treatment decreased T cell arrest. While blockade of antigen recognition directly impairs CD4^+^ T cell/APC interactions, decreased CD8^+^ T cell arrest suggests an indirect effect. Since CD4^+^/APCs clusters may attract CD8^+^ T cells (39), CD8^+^ T cells might be more likely to contact APCs once they are or have already been in contact with CD4^+^ T cells, as described in LN (19, 20). This may explain why CD4^+^/DC/CD8^+^ clusters could be frequently detected by confocal experiments. However, further experiments would be required to evaluate the importance of three-cell type interactions in help delivery.

Finally, anti-MHCII antibody treatment at a time when antigen-driven activation of HA-specific T cells in the pLN has already occurred and effector T cells have already infiltrated the pancreas affected the expression of markers linked to effector functions in HA-specific CD8^+^ T cells. This supports a pivotal role for continued CD4^+^ T cell help, mediated by APCs, in maintaining CD8^+^ T cell killer efficiency in the pancreas. CTLs generated with CD4 help in pLN may thus not be programmed to retain effector functions in the pancreas and need continued help for optimal functionality. Consistent with antigen-dependent CD4^+^ T cell extravasation in the pancreas facilitated by DCs (32), anti-MHCII treatment impaired CD4^+^ T cell recruitment. Interestingly, CD8^+^ T cell numbers were also affected, reflecting the role of CD4^+^ T cells in CD8^+^ T cell recruitment and/or proliferation in the pancreas (16). The delay in blood glucose rise and later stabilization of both blood glucose levels and general health thus likely reflect a combined effect of the reduced effector cell infiltration and the reduced ability of helpless effector CD8^+^ T cells to sustain beta cell destruction.

Precise mechanisms underlying help delivery upon antigen recognition in the pancreas still need further investigation. In LNs, CD40-mediated licensing of DCs by CD4^+^ T cells induces the expression of co-stimulatory molecules and constitutes the main pathway of CD4 help for naive CD8^+^ T cell priming (40). However, requirements for effector CD8^+^ T cell restimulation are likely to be less stringent. Supporting this, pancreatic DCs from anti-MHCII-treated mice displayed similar levels of co-stimulatory molecules as controls (our unpublished observations). Alternatively, the facts that antitumor CTL rely on IL-2 and IFNγ for tumor rejection (16) and that pancreatic HNT CD4^+^ T cells were able here to secrete IFNγ and IL-2 in response to antigen suggest that these cytokines may be involved in providing help to clone 4 CD8^+^ T cells. A tantalizing prospect is that DCs may serve as a platform for delivering these cytokines.

In summary, we reveal a pivotal role for continued CD4 help in maintaining CD8^+^ T cell effector function in the pancreas and in autoimmune diabetes progression and thus unveil an unprecedented role for cooperation between effector CD4^+^ and CD8^+^ T cells that may have important implications for the design of new therapeutic strategies against T1D.

## Ethics Statement

All animal studies were conducted according to the European guidelines for animal welfare and approved by the Institutional Animal Care and Use Committee (CEEA-LR-1190 and CEEA-LR-12163) and the French Ministry of Agriculture (APAFIS#3874).

## Author Contributions

GE-C, JH, and MS designed experiments; GE-C, CS, and MS performed experiments; GE-C, PF, JH, and MS analyzed data; TS provided reagents; and PM, JH, and MS wrote the manuscript.

## Conflict of Interest Statement

The authors declare that the research was conducted in the absence of any commercial or financial relationships that could be construed as a potential conflict of interest.
