# Supplementary material for: CD4+ T Helper Cells Play a Key Role in Maintaining Diabetogenic CD8+ T Cell Function in the Pancreas
Source: Front Immunol. 2018 Jan 18;8:2001. doi: 10.3389/fimmu.2017.02001 (PMC5778106; doi:10.3389/fimmu.2017.02001)
Supplement: Supplementary file 7 [file Data_Sheet_1.docx]

**SUPPLEMENTARY MATERIALS**

**SUPPLEMENTARY FIGURES**

**
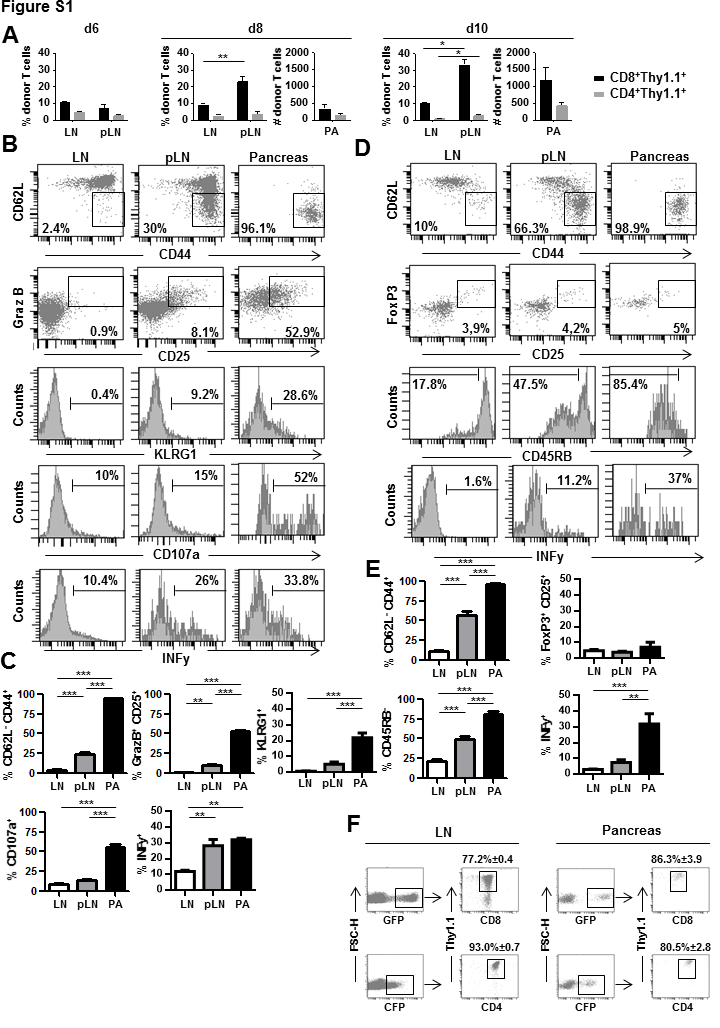
**

**Figure S1. Activation and phenotype of diabetogenic HA-specific CD8^+^ and CD4^+^ T cells in InsHA-mCherry mice.** Irradiated InsHA-mCherry mice were adoptively transferred with Clone 4-GFP CD8^+^ and HNT-CFP CD4^+^ T cells and single cell suspensions from pancreas, LN and pLN were analyzed by FACS. LN: lymph nodes; pLN, pancreatic lymph nodes; PA: pancreas. **A**) At the indicated days post-transfer, donor CD8^+^ and CD4^+^ T cells were detected by virtue of the expression of the Thy1.1 allele gating in living singlet lymphocytes,. Graphs depict percentages or event counts as mean ± SEM (n = 4-5 mice/group, one representative experiment out 2 to 7, Mann-Whitney). When gating on Thy1.1^+^ cells, CD4^+^ and CD8^+^ cells segregate into unique populations (data not shown). **B-C**) Phenotype and effector function potential of donor Clone 4 Thy1.1^+^ CD8^+^ T cells on day 8 post-transfer. Plots in (B) represent an individual representative mouse, while graphs in (C) represent mean ± SEM (n = 3-4 mice, one representative experiment out of four, Mann-Whitney). GrazB: GranzymeB. **D-E**) Phenotype and effector function potential of donor Thy1.1^+^ CD4^+^ HNT T cells. Plots in (D) represent an individual representative mouse, while graphs in (E) represent mean ± SEM (n = 3-4 mice, one representative experiment out of four, Mann-Whitney). **F**) Donor Clone 4-GFP and HNT-CFP T cells were detected by virtue of GFP or CFP fluorescence respectively in LN and pancreas. Percentages of CD8^+^ Thy1.1^+^ cells gating in GFP or CD4^+^ Thy1.1^+^ cells gating on CFP are represented (mean ± SEM, n = 4-5 mice from one out two independent experiments).


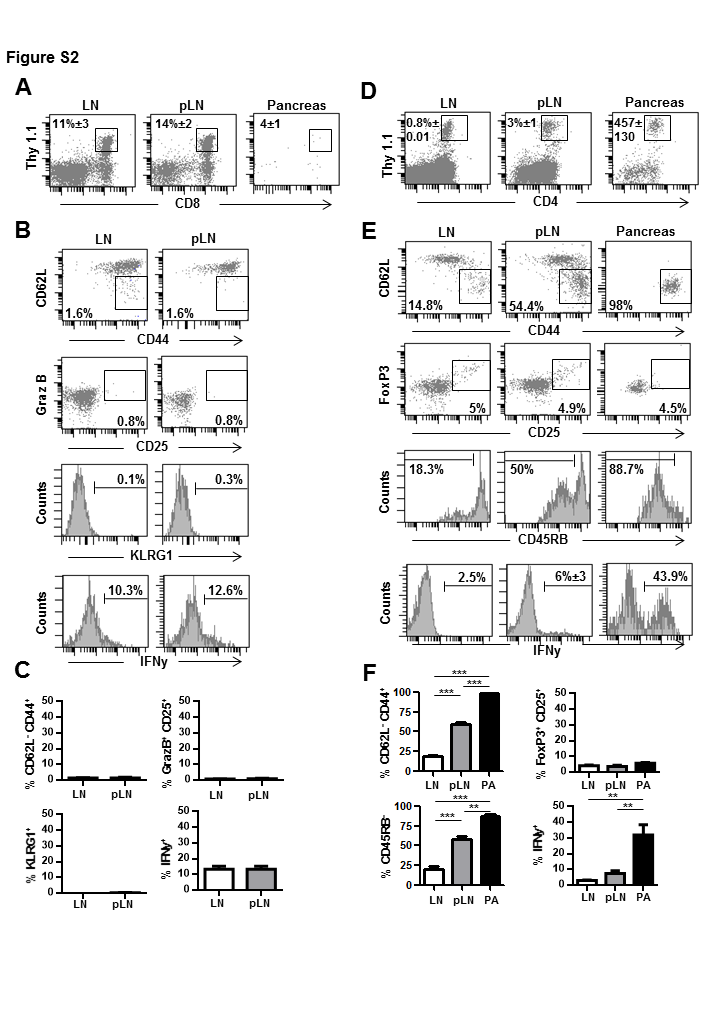


**Figure S2. Activation and phenotype of HA-specific CD8^+^ and CD4^+^ T cells transferred separately into InsHA-mCherry mice.** Irradiated InsHA-mCherry mice were adoptively transferred with either Clone 4-GFP CD8^+^ (A-C) or HNT-CFP CD4^+^ T cells (D-F) and single cell suspensions from pancreas, LN and pLN were analyzed by FACS on day 8-10 after transfer. **A**) Donor CD8^+^ T cells were detected by virtue of the expression of the Thy1.1 allele, gating in living singlet lymphocytes. Plots are from a single representative mouse, and values indicate percentages or event counts in the depicted gates as mean ± SEM (n = 3-5 mice/group, one representative experiment out of six). **B-C**) Phenotype and effector function potential of donor Clone 4 Thy1.1^+^ CD8^+^ T cells. Plots of representative individual InsHA-mCherry mice (B). Graphs represent mean ± SEM (n = 3 mice, one representative experiment out of three, Mann-Whitney) (C). **D**) Donor CD4^+^ T cells were detected by virtue of the expression of the Thy1.1 allele, gating in living singlet lymphocytes. Plots are from a single representative mouse and values indicate percentages or event counts in the depicted gates as mean ± SEM (n = 3-5 mice/group, one representative experiment out of six). **E-F**) Phenotype and effector function potential of donor HNT Thy1.1^+^ CD4^+^ T cells. Plots of representative individual InsHA-mCherry mice (E). Graphs represent mean ± SEM (n = 3 mice, one representative experiment out of four, Mann-Whitney) (F).


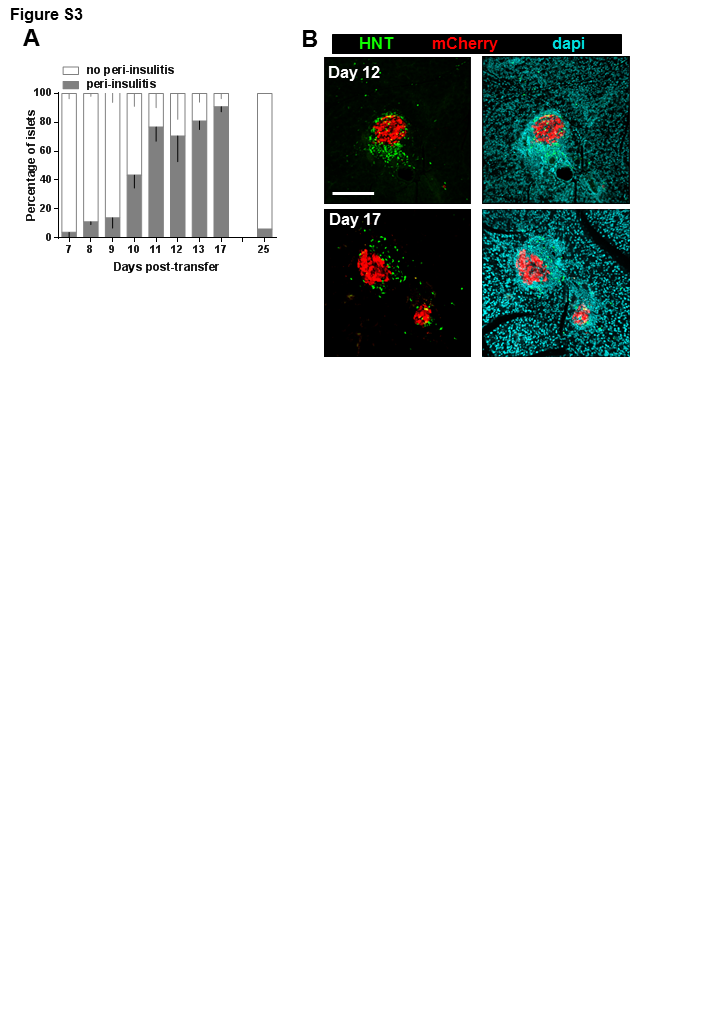


**Figure S3. Kinetics and pattern of HNT-CFP CD4^+^ T cell infiltration in the pancreas of InsHA-mCherry mice. A**) Percentage of infiltrated islets in pancreata from irradiated InsHA-mCherry mice as function of days post-transfer of HNT-GFP CD4^+^ T cells (n = 2-3 mice/day, > 60 islets/mouse). Values represent mean ± SEM. **B**) Representative confocal images of pancreas from mice described in panel (A), at day 12 and 17 post-transfer of HNT-GFP CD4^+^ T cells (scale: 200 µm, Z-projection of 20 µm).

**
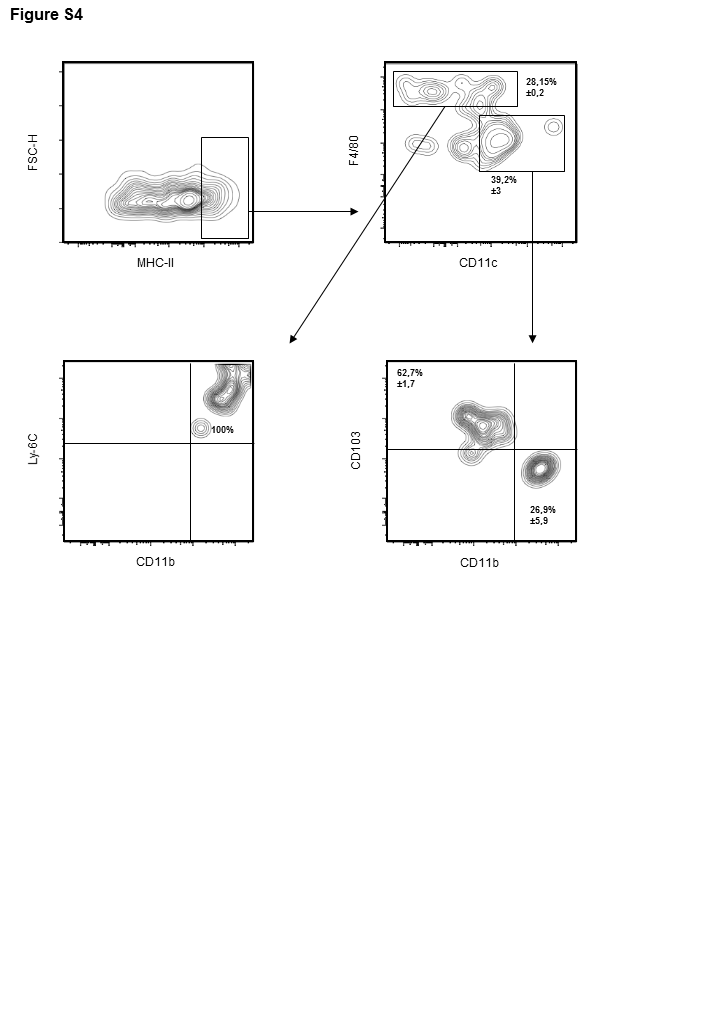
 Figure S4. Characterization of recruited APCs in the pancreas of pre-diabetic InsHA-mCherry mice.** Irradiated InsHA-mCherry mice adoptively transferred with Clone 4 CD8^+^ and HNT CD4^+^ T cells were sacrificed at day 10 post-transfer. Single cell suspensions from collagenase-digested pancreas were analyzed by flow cytometry gating on living singlet leukocytes. After exclusion of CD3^+^ CD19^+^ and NKp46^+^ cells, MHC II^hi^ cells were considered as APCs (upper left panel). Expression of F4/80 and CD11c was then used to discriminate macrophages and DCs respectively (upper right panel). F4/80^+^ macrophages were further characterized by the expression of Ly-6C and CD11b (lower left panel). CD11c^hi^ DCs were further characterized by the expression of CD103 and CD11b (lower right panel). Percentages of different subpopulations of APCs correspond to mean ± SEM of one representative experiment out of three.

**
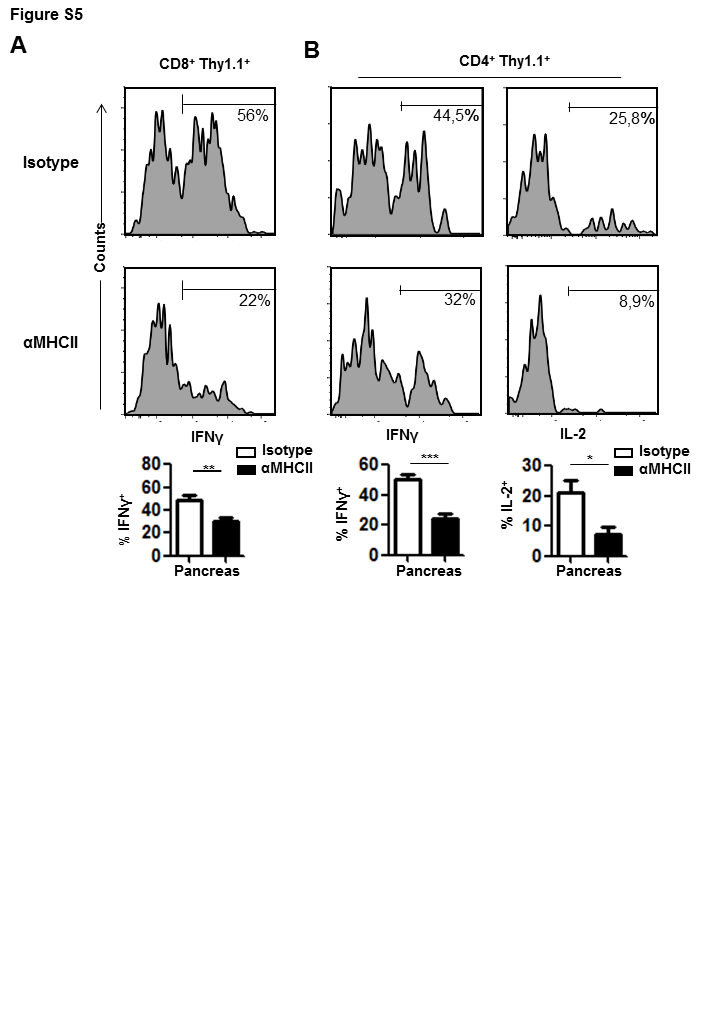
**

**Figure S5. MHC class II blockade decreases HA-specific CD8^+^ and CD4^+^ T cell effector potential in the pancreas.** Groups of irradiated InsHA-mCherry mice adoptively transferred with Clone 4-GFP CD8^+^ and HNT-CFP CD4^+^ T cells were treated with either anti-MHC II or isotype matched control mAbs on days 8 and 9. Mice were sacrificed at day 10 and lymphocytes from the pancreas were restimulated to assess cytokine production by FACS. **A**) INFγ production by donor Clone 4 CD8^+^ Thy1.1^+^ T cells in the pancreas of representative individual mice. Graph depicts percentage of IFNγ secreting donor CD8^+^ T cells in the pancreas. Values correspond to mean ± SEM of one representative experiment out of two (n = 3-5 mice/group). **B**) IL-2 and IFNγ production by donor HNT CD4^+^ Thy1.1^+^ T cells in the pancreas of representative individual mice. Graphs depict percentages of IL-2 and IFNγ secreting donor CD4^+^ T cells in the pancreas. Values in graphs correspond to mean ± SEM of two independent experiments (n = 4-10 mice/group).**SUPPLEMENTAL VIDEOS**

**Video S1. Intra-vital imaging of islet antigen-specific CD4^+^ and CD8^+^ T cells in the pancreas.** *In vivo* recording of T cell motility around an infiltrated islet in the pancreas of an InsHA-mCherry mouse transferred with HNT-CFP CD4^+^ and Clone 4-GFP CD8^+^ T cells at day 8 post-transfer. Movie rate: 7 frames/s. Total elapsed time: 18.75 min. Green: GFP, blue: CFP, red: mCherry. Image size: 250 × 250 μm, 204 µm z-projection.

**Video S2. Islet antigen-specific CD8^+^ T cells contact beta cells.** *In vivo* recording in a pancreas of an InsHA-mCherry mouse transferred with HNT-CFP CD4^+^ and Clone 4-GFP CD8^+^ T cells at day 8 post-transfer. Bottom circle: one Clone 4-GFP CD8^+^ T cell interacting with a beta cell; middle circle: several Clone 4-GFP CD8^+^ T cells interacting with a beta cell. Green: GFP, blue: CFP, red: mCherry. Movie rate: 7 frames/s. Total elapsed time: 18.75 min. Image size: 300 × 300 μm, 204 µm z-projection.

**Video S3. Composition and severity of pancreatic T cell infiltrates is heterogeneous.** *In vivo* recordings in different areas of a single pancreas of an InsHA-mCherry mouse transferred with HNT-CFP CD4^+^ and Clone 4-GFP CD8^+^ T cells at day 8 post-transfer. First video: mild infiltration, characterized but high CFP/GFP ratio. Total elapsed time: 15.5 min. Second video: severe infiltration, characterized by lower CFP/GFP ratio. Total elapsed time: 14.5 min. Islets are circled. Green: GFP, blue: CFP, red: mCherry. Movie rate: 7 frames/s. Image size: 600 × 600 μm, 177 µm z-projection.

**Video S4. Islet antigen-specific CD8^+^ T dividing *in vivo*.** *In vivo* recording showing an example of a dividing Clone 4-GFP CD8^+^ T cell, in the pancreas of an InsHA-mCherry mouse transferred with HNT-CFP CD4^+^ and Clone 4-GFP CD8^+^ T cells at day 8 post-transfer. Movie rate: 7 frames/s. Total elapsed time: 22.5 min. Green: GFP, blue: CFP. Image size: 150 × 150 μm, 100 µm z-projection.

**Video S5. T cell motility in heavily infiltrated exocrine tissue.** *In vivo* recording of a representative heavily infiltrated area in the pancreas of an InsHA-mCherry mouse transferred with HNT-CFP CD4^+^ and Clone 4-GFP CD8^+^ T cells at day 8 post-transfer. Movie rate: 7 frames/s. Total elapsed time: 15.25 min. Green: GFP, blue: CFP. Image size: 640 × 640 μm, 177 µm z-projection.

**Video S6. Anti-MHC class II mAb treatment reduced CD4^+^ T cells arrest.** *In vivo* recordings showing T cell motility 1-2 h following i.v. injection of isotype control mAb (left) and anti-MHC class II mAb (right), in the pancreas of InsHA-mCherry mice transferred with HNT-CFP CD4^+^ and Clone 4-GFP CD8^+^ T cells, at day 8 post-transfer. Movie rates: 7 frames/s. Total elapsed time: 16 min. Green: GFP, blue: CFP. Image size: 300 × 300 μm, 190 µm z-projection.
